# Obituary

**Published:** 1891-07

**Authors:** 


### Obituary.

Died, at his home in Springfield, Ohio, June 4th, Dr. Frank C. Runyan after a long and painful illness. He has been in feeble health for over a year. The immediate cause of his death was the amputation of his leg; the shock being too great for his enfeebled condition. There has been, for months, necrosis of the bone of the limb.

Dr. Runyan has been a leading member of the dental profession in Central Ohio for many years. He was a man of definite and pronounced views and opinions, and always prompt to advocate and maintain what he thought to be right. He has been for many years an active member of the Ohio State Dental Society; he always took an interest in all that pertained to its welfare and prosperity. He was a graduate of the Ohio College of Dental Surgery, taking his degree from this institution in 1872, since which time he has been engaged in the practice of his profession in the city of his home. He was one of the men whom the profession can ill afford to spare. He leaves a wife, Mrs. Georgie D. Runyan, a literary writer of marked merit. The family will have the warm sympathy of all who knew Dr. Runyan.

